# Demonstration of a Simple Epitope Tag Multimerization Strategy for Enhancing the Sensitivity of Protein Detection Using *Drosophila* vAChT

**DOI:** 10.1534/g3.119.400750

**Published:** 2019-11-25

**Authors:** Kole V. Tison, Hannah M. McKinney, R. Steven Stowers

**Affiliations:** Department of Cell Biology and Neuroscience, Montana State University, Bozeman MT 59717

**Keywords:** Drosophila, epitope tag, cholinergic, multimerization, vAChT

## Abstract

The expression and distribution of a protein can provide critical information about its function in a cell. For some neuronal proteins this information may include neurotransmitter (NT) usage and sites of NT release. However, visualizing the expression of a protein within a given neuron is often challenging because most neurons are intricately intermingled with numerous other neurons, making individual neuronal expression difficult to discern, especially since many neuronal genes are expressed at low levels. To overcome these difficulties for the *Drosophila* vesicular acetylcholine transporter (vAChT), attempts were made to generate conditional *Drosophila* vAChT alleles containing two tandem copies of epitope tags. In the course of these attempts, a strategy for multimerizing DNA repeats using the Gibson cloning reaction was serendipitously discovered. Attempts at optimization routinely yielded six or seven copies of MYC and OLLAS epitope tag coding sequences, but occasionally as many as 10 copies, thus potentially enhancing the sensitivity of protein detection up to an order of magnitude. As proof-of-principle of the method, conditionally expressible genome-edited 7XMYC-vAChT and 6XOLLAS-vAChT were developed and characterized for conditionality, synaptic vesicle specificity, and neurotransmitter specific-expression. The utility of these conditional vAChT variants was demonstrated for cholinergic neurotransmitter phenotyping and defining the polarity of cholinergic neurons, important information for understanding the functional role of neurons of interest in neural circuits and behavior. The repeat multimerization method is effective for DNA repeats of at least 56 bp and should be generally applicable to any species.

Knowledge of the spatial distribution of a protein within a cell is instrumental to understanding its function. This is nowhere more apparent than in the nervous system. Factors that limit the ability to ascertain the distribution of a protein in a neuron include the sensitivity of protein detection and the extensive intermingling of neurons with each other. For broadly expressed proteins, this latter issue is particularly problematic as axons and dendrites from hundreds of neurons often converge with each other, thus making it impossible to determine with any certainty whether observed expression of a protein in densely packed neuropil comes from any specific neuron. A solution to this problem is to conditionally express a protein of interest at endogenous levels only in small subsets of neurons or even in single neurons. This approach has been taken for several Drosophila neuronal proteins including the active zone protein Brp (Chen *et al.* 2014), the vesicular neurotransmitter transporter for acetylcholine vAChT (Pankova and Borst 2017), and the synaptic vesicle (SV)-specific protein Rab3 (Williams *et al.* 2019). However, this strategy often exposes the problem of sensitivity, especially for proteins with low endogenous levels of expression as is common with many neuronal proteins.

Here we describe a simple strategy for epitope tag multimerization that involves placing two tandem copies of an epitope tag coding sequence at a Gibson junction. This approach routinely yields at least six copies, but sometimes as many as 10 copies, of commonly used epitope tags. Using this strategy, the sensitivity of protein detection can thus be enhanced up to an order of magnitude. We demonstrate the utility of the method for conditionally expressible variants of Drosophila vAChT that utilize 7XMYC and 6XOLLAS epitope tags, but the strategy is generally applicable to any protein in any species.

## Materials and Methods

### Plasmid construction

The *pCFD4-vAChT* double guide RNA plasmid was generated as previously described (Port *et al.* 2014) and includes guide RNA sequences cagagaagagtacaaaca and agcaaccgagaacagtga. The donor plasmids were assembled using NEBuilder HiFi (New England Biolabs). Two tandem copies of the epitope tag sequence were present on complementary oligonucleotides that formed the junction where repeat multimerization occurred as shown in Figure 2C. Donor plasmids were also constructed using NEBuilder HiFi in the vector *pHSG298* (Takara Biosciences). The complete sequences of all donor plasmids are shown in Supplemental Information.

The *20XUAS-DSCP-FLP* expression plasmid was assembled by Gateway MultiSite cloning (Petersen and Stowers 2011; Shearin *et al.* 2013). The component entry clones were *L1-20XUAS-DSCP-R5* (Williams *et al.* 2019), *L5-FLP-L2*, and *pDESTp10* (Shearin *et al.* 2013). The *L5-FLP-L2* entry clone was newly generated using previously described methods (Petersen and Stowers 2011).

The sequences of the oligonucleotides containing the MYC epitope tag coding sequences used to generate the DNA fragments constituting the Gibson junction with overlapping MYC repeats are indicated below (oligonucleotides with which each was paired not shown). The MYC repeat sequences are capitalized and bold, the linker sequences are lowercase, and the vAChT locus-specific sequences are capitalized but not bold. The oligonucleotide sequences used to generate the DNA fragments containing the OLLAS repeats found in *FRT-STOP-FRT-6XOLLAS-vAChT* are identical except OLLAS epitope tag coding sequences were substituted for the indicated MYC sequences.

#### vAChTMYCF2:

5′**GAGCAGAAGCTGATCAGCGAGGAAGATCTG**ggcggatctggc**GAGCAGAAGCTGATCAGCGAGGAAGATCTG**ggcggatctggcGCCTCATTCCAAATACCTGTTATCAACCTG3′

#### vAChTMYCR2:

5′**CAGATCTTCCTCGCTGATCAGCTTCTGCTC**gccagatccgcc**CAGATCTTCCTCGCTGATCAGCTTCTGCTC**CATTTTGGTTGCAATTAATTAATTTCAATTGCTGA3′

### Transgenic fly strains

The previously described *N-syb-GAL4* expression clone (Williams *et al.* 2019) was inserted at landing site *JMK22C*. The *20XUAS-DSCP-FLP* expression clone was inserted at landing site *VK00005*.

### Genome editing

The guide RNA *pCFD4-vAChT* was co-injected with donor plasmids into embryos of strain *nos-Cas9 TH_attP40* (Ren *et al.* 2013) by Bestgene, Inc. The ∼50 adults for each construct that typically survived the injections were crossed to a third chromosome balancer strain in combinations of 2-3 males to 10 balancer females or 5-6 females to 5-6 balancer males. For each donor construct, ∼150 individual males from the balancer cross were complementation tested against the existing *vAChT^1^* allele as the desired genome edit of each donor construct was expected to produce a null vAChT allele due to the presence of the upstream STOP cassette. This typically resulted in 5-10 failed complementation crosses per donor construct, most of which were independent since they originated from separate balancer crosses. Male progeny from failed complementation crosses were subsequently crossed to a third chromosome balancer strain to establish stable lines. Germline excisions were generated as previously described (Williams *et al.* 2019).

### Immunostaining

Immunostaining was performed as previously described (Certel and Thor 2004). Primary antibodies and dilution factors: The SYN (3C11) mAb 1:50 developed by E. Buchner (Klagges *et al.* 1996) was obtained from the Developmental Studies Hybridoma Bank, created by the NICHD of the NIH and maintained at The University of Iowa, Department of Biology, Iowa City, IA 52242; Rabbit Abfinity anti-GFP (Thermo-Fisher) 1:500, Mouse anti-GFP 3E6 (Thermo-Fisher) 1:200, Rat anti-mCherry 16D7 (Thermo-Fisher) 1:500, Rabbit anti-mCherry (Abcam ab213511) 1:500, Mouse anti-mCherry 1:300 (Biorbyt orb256058); Rat anti-HA 3F10 (Sigma-Aldrich) 1:100; Rat anti-OLLAS L2 1:200 (Novus); Rabbit anti-MYC 9E10 (Novus) 1:400. Secondary antibodies and dilution factors: Donkey anti-Mouse Alexa 488 (Jackson Immunoresearch 715-546-151) 1:400; Goat anti-Rabbit Alexa 488 (Thermo-Fisher A32731) 1:400; Donkey anti-Rat Alexa 488 (Jackson 712-546-153) 1:400; Goat anti-Rabbit JF549 (Novus NBP1-72732JF549) 1:200; Donkey anti-Mouse JF549 (Novus NBP1-75119JF549) 1:200; Goat anti-Rat JF549 (Novus NBP1-75398JF549) 1:200; Goat anti-Rabbit JF646 (NBP1-72732JF646) 1:200; Donkey anti-Mouse JF646 (Novus NBP1-75119JF646) 1:200, Goat anti-Rat JF646 (Novus NBP1-75398JF646) 1:200.

### Fly strains

Stocks from the Bloomington Drosophila Stock Center (NIH P40OD018537) were used in this study. Previously described fly strains: *UAS-CD8-mCherry* (BDSC# 27392); *nos-GAL4* (BDSC # 25394) (Tracey *et al.* 2000); UAS*-DSCP-B2* (Williams *et al.* 2019); LH2094 *VT006486-p65ADZp* (*attP40*); *VT008489-ZpGDBD* (*attP2*) (Dolan *et al.* 2019); MBON-6 MB434B *R30E08-p65ADzp* (*attP40*); *R53C10-ZpGdbd* (*attP2*); LH1900 *R17A04-p65ADZp* (*attP40*); *VT041432-ZpGDBD* (*attP2*) (Aso *et al.* 2014, Dolan *et al.* 2019); SS02702 *R67A06-p65ADZp* (*attP40*); *R11F03-ZpGdbd* (*attP2*) (Robie *et al.* 2017); LPi4-3 *R38G02-p65ADZp* (*attP40*); *R24A07-ZpGdbd* (*attP2*) (Klapoetke *et al.* 2017); LPLC1 *R64G09-p65ADZp* (*attP40*); *R37H04-ZpGAL4DBD* (*attP2*) (Wu *et al.* 2016); MBON-22 *R64F07-p65ADZp* (*su(Hw)attP8*); *R57C10-ZpGdbd* (*attP2*) (Aso *et al.* 2014).

### Data availability

Complete sequences of entry clones are available upon request. Complete sequences of donor plasmids are shown in Supplemental Information. Fly strains original to this publication will be deposited at the Bloomington Drosophila stock center or will be made available upon request. Entry clones and donor plasmids will be deposited at Addgene or will be made available upon request. Supplemental material available at figshare: https://doi.org/10.25387/g3.9882908.

## Results

### Discovery and attempted optimization of the multimerization phenomenon

**Figure 1:**
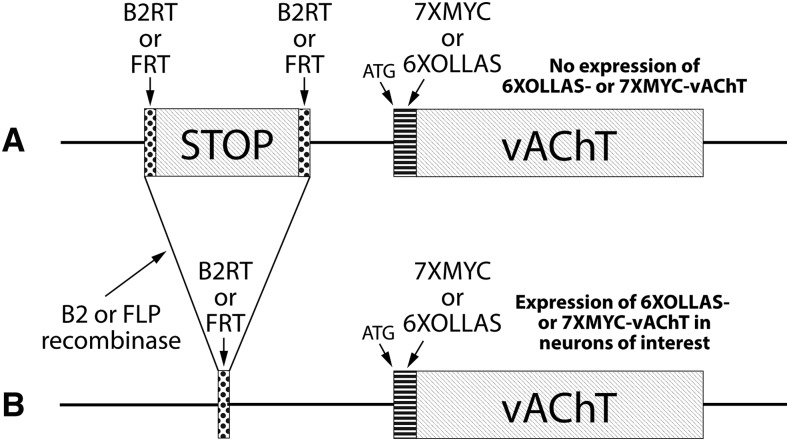
Strategic design of conditional epitope-tagged Drosophila vAChT. A) A *B2RT* or *FRT*-flanked transcription STOP cassette was inserted upstream of the vAChT coding sequence and 7XMYC or 6XOLLAS epitope tag coding sequences were fused to the amino-terminus of vAChT. Prior to STOP cassette excision, there is no expression of epitope tagged-vAChT. B) After expression of the B2 or FLP recombinases in neurons of interest, typically using a GAL4 driver and *UAS-B2* or *UAS-FLP* transgenes, the STOP cassette is excised and epitope tagged variants of vAChT are expressed in cholinergic neurons. All genome edits were carried out at the endogenous *vAChT* locus.

Previous results using the conditionally expressible *FRT-STOP-FRT-HA-vAChT* (Pankova and Borst 2017) with a single copy of the HA epitope tag revealed sub-optimal sensitivity of detection in small neuronal subsets (Williams *et al.* 2019). To generate a cholinergic SV marker with a higher sensitivity of detection and distinct epitope tags, donor constructs were designed to contain two copies of the epitope tags OLLAS or MYC at the amino-terminus of vAChT for CRISPR/Cas9 genome editing (Figure 1). To allow compatibility with simultaneous use of multiple recombinases, both *FRT* and *B2RT* versions of each were designed. The strategy for conditionality was the same as previously reported for *FRT-STOP-FRT-HA-vAChT* where an upstream transcription STOP cassette (Nern *et al.* 2011) was flanked by recombinase target sites. Prior to excision of the STOP cassette, the epitope-tagged vAChT is not expressed (Figure 1A), but after selective expression of either the FLP or B2 recombinases (typically using a GAL4 driver), the OLLAS or MYC-tagged tagged vAChT is specifically expressed in cholinergic neurons of interest (Figure 1B).

**Figure 2:**
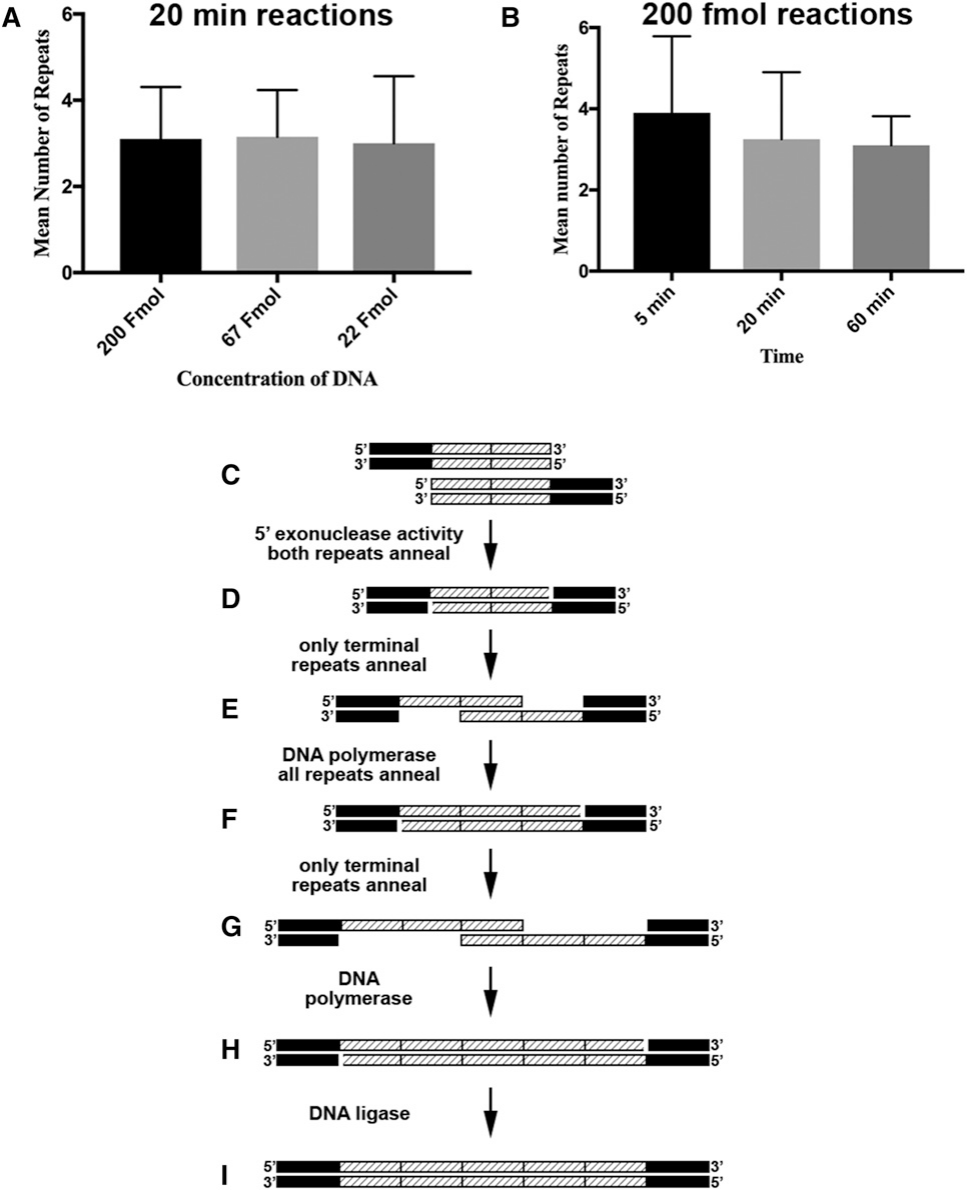
Attempted optimization and potential mechanism for Gibson reaction-mediated DNA repeat multimerization. A) Optimization with respect to DNA substrate concentration. Reaction times were 20 min. B) Optimization with respect to time. All reactions contained 200 fmol of substrate DNA. C) Substrates for Gibson-mediated DNA repeat multimerization are two double stranded DNA fragments containing two overlapping tandem copies of the repeat sequence. D) After 5′ exonuclease activity and annealing of both repeats. E) Reconfiguration of annealing such that only the terminal repeats anneal. F) The result of DNA polymerase activity from the configuration shown in E. G) Reconfiguration of annealing shown in F such that only the terminal repeats anneal. H) The result of DNA polymerase activity from the configuration shown in G. I) A stable double-stranded DNA molecule results after DNA ligase activity of H. Sucecssive rounds of reconfigured annealing occurring after DNA synthesis, but before ligation, could yield higher numbers of repeats.

The donor constructs were assembled using NEB HiFi/Gibson cloning (hereafter Gibson) (Gibson *et al.* 2009; Gibson *et al.* 2010) such that the 2XOLLAS or 2XMYC epitope tags were at a Gibson junction (Figure 2C). Unexpectedly, in screening individual clones of the donor constructs via restriction analysis it was noticed that the restriction fragments containing the presumptive 2XOLLAS or 2XMYC epitope tag coding sequences were larger than expected for some clones. Sequence analysis of these clones revealed the larger fragment sizes were due to the presence of more than the expected two copies of the epitope tag coding sequences.

For the *B2RT-STOP-B2RT-2XMYC-vAChT* donor construct, attempts were made to optimize the multimerization phenomenon by varying the quantity of substrate DNA and length of time of the Gibson reactions. For optimization of DNA quantity, *Xho* I/*Pst* I restriction digests for 20 independent clones resulting from 20-minute Gibson reactions using 200 (Figure S1A), 67 (Figure S1B), or 22 fmol (Figure S1C) of substrate DNA are shown. These restriction digests yielded constant bands of 5087, 1952, and 586 bp as well as variable bands from 642 bp (2XMYC) to 852 bp (7XMYC) in 42bp increments. The flexible linker GGSG was included between each MYC repeat to maximize the antigenic availability of the epitope tag and minimize the chances the tag alters the function of the vAChT protein. For each reaction condition, the MYC repeat number was quantitated as indicated for 200 (Figure S2A), 67 (Figure S2B), and 22 fmol (Figure S2C). Each reaction condition yielded at least one clone containing a 7XMYC repeat (verified by sequencing). A summary of the results showed no significant difference of mean repeat number among the different quantities of substrate DNA tested that varied over the ninefold concentration range tested (Figure 2A).

An attempt to optimize the multimerization phenomenon with respect to reaction time was also performed. For optimization of Gibson reaction time, *Xho* I/*Pst* I restriction digests for 20 independent clones resulting from 200 fmols of substrate DNA for five (Figure S1D), 20 (Figure S1E), or 60-minute (Figure S1F) Gibson reactions are shown. For each reaction condition, the MYC repeat number was quantitated as indicated for five (Figure S2D), 20 (Figure S2E), and 60 min (Figure S2F). Maximum MYC repeat numbers (sequence verified) were seven for the five-minute reaction, 10 for the 20-minute reaction, and five for the 60-minute reaction. The mean number of repeats was highest for the five-minute reaction and decreased with time, although these differences were not statistically significant between each other (Figure 1B) or between the conditions tested for substrate DNA concentration. Additional statistical information for attempted optimization with respect to DNA quantity and length of Gibson reaction time can be found in Table S1. Unfortunately, the clone containing 10XMYC was determined to have a single base pair deletion in one of the repeats, thus shifting the open reading frame and making it unusable. No other errors were detected among several independent 7XMYC and 6XOLLAS clones that were sequenced, thus indicating this method of DNA repeat multimerization only rarely produces mutations.

A Gibson reaction for the designed *FRT-STOP-FRT-2XOLLAS-vAChT* clone using 200 fmol of substrate DNA for 20 min yielded a maximum of 6XOLLAS repeats. This result shows the repeat multimerization phenomenon is generalizable and not specific for either the OLLAS or MYC epitope tag coding sequences. *The FRT-STOP-FRT-7XMYC-vAChT* and *B2RT-STOP-B2RT-6XOLLAS-vAChT* donor constructs were assembled by restriction cloning between *B2RT-STOP-B2RT-7XMYC-vAChT* and *FRT-STOP-FRT-6XOLLAS-vAChT*.

### Mechanism of Gibson-mediated repeat multimerization

What is the mechanism for the observed Gibson-mediated DNA repeat multimerization phenomenon? Gibson reactions utilize three enzymes: 1) a 5′ to 3′ DNA exonuclease; 2) a DNA polymerase; and 3) a DNA ligase. The initial substrates for the Gibson reaction at the repeat junction were two identical double-stranded overlapping repeats (Figure 2C). A potential mechanism is as follows. From the starting substrates, 5′ to 3′ exonuclease activity first cleaves both repeats on one strand of each double-stranded DNA. In this scenario, both repeats on the remaining strands could anneal (Figure 2D) or, alternatively, only the terminal repeats anneal (Figure 2E). In the latter scenario, DNA polymerase activity would result in three copies of the repeat sequence (Figure 2F). If, however, before ligation occurs, the repeats reconfigure such that only the terminal repeats are annealed (Figure 2G), DNA polymerase activity could add two additional repeats to each strand (Figure 2H). Subsequent DNA ligation would result in five tandem copies of the repeat sequence (Figure 2I). Successive rounds of reconfigured annealing (similar to Figure 2G) after synthesis of the repeats but prior to DNA ligation would yield additional copies of the repeat sequence.

### Assessment of 7XMYC-vAChT for conditionality and SV specificity

Each of the four conditional, epitope-tagged vAChT variants (Table 1) was integrated into the endogenous vAChT genomic locus via CRISPR/Cas9 genome editing (Gratz *et al.* 2013; Ren *et al.* 2013). This ensures conditional expression of each vAChT variant will recapitulate the endogenous neuronal distribution of vAChT at endogenous vAChT expression levels.

**Table 1:** Novel Fly Strains

*B2RT-STOP-B2RT-6XOLLAS-vAChT*
*FRT-STOP-FRT-6XOLLAS-vAChT*
*B2RT-STOP-B2RT-7XMYC-vAChT*
*FRT-STOP-FRT-7XMYC-vAChT*

To assess whether *FRT-STOP-FRT-7XMYC-vAChT* exhibits a SV-specific distribution, the STOP cassette was excised both pan-neuronally and in the germline and compared to expression of the SV-specific protein Synapsin (Klagges *et al.* 1996; Fernández-Chacón and Südhof 1999). Pan-neuronal excision of the STOP cassette using *N-syb-GAL4* to drive FLP recombinase expression revealed a neuropil distribution of 7XMYC-vAChT (Figure S3A_2_) that closely resembles that of endogenous Synapsin (Figure S3A_3_) as indicated in the overlay (Figure S3A_4_). This expression pattern is noticeably distinct from the pan-neuronal distribution of plasma membrane marker CD8-mCherry (Figure S3A_1_). Similarly, germline excision of the STOP cassette shows a neuropil distribution (Figure S3B_2_) that closely resembles Synapsin (Figure S3B_3_) as revealed in the overlay (Figure S3B_4_). An overlay of the stack comprising Figure S3B is presented as a movie to enable visualization of individual slices (Fig S4). No expression of 7XMYC-vAChT was observed in the absence of a GAL4 driver (Figure S3C_2_), thus indicating *FRT-STOP-FRT-7XMYC-vAChT* is conditional and not constitutively expressed prior to STOP cassette excision.

Similar results were observed for *B2RT-STOP-B2RT-7XMYC-vAChT*. Pan-neuronal STOP cassette excision using *N-syb-GAL4* resulted in a neuropil distribution of 7XMYC-vAChT (Figure S3D_2_) that closely matches that of endogenous Synapsin (Figure S3D_3_), as indicated in the overlay (Figure S3D_4_), that is distinct from CD8-mCherry (Figure S3D_1_). Germline excision of the STOP cassette also reveals a neuropil distribution of 7XMYC-vAChT (Figure S3E_2_) nearly indistinguishable from Synapsin (Figure S3E_3_) as indicated in the overlay (Figure S3E_4_). No expression of 7XMYC-vAChT was observed in the absence of a GAL4 driver (Figure S3F_2_), thus indicating *B2RT-STOP-B2RT-7XMYC-vAChT* is conditional and not constitutively expressed prior to STOP cassette excision. MYC immunostaining in a *yw* control brain revealed almost no background (Figure S3G_2_) indicating the anti-MYC antibody does not recognize endogenous Drosophila antigens.

### Assessment of 6XOLLAS-vAChT for conditionality and specificity

To assess whether *FRT-STOP-FRT-6XOLLAS-vAChT* exhibits a SV-specific distribution, the STOP cassette was excised both pan-neuronally and in the germline and compared to expression of the SV-specific protein Synapsin. Pan-neuronal excision of the STOP cassette using *N-syb-GAL4* to drive FLP recombinase expression revealed a neuropil distribution of 6XOLLAS-vAChT (Figure S5A_2_) that closely resembles that of endogenous Synapsin (Figure S5A_3_) as indicated in the overlay (Figure S5A_4_). This expression pattern is noticeably distinct from the pan-neuronal distribution of plasma membrane marker CD8-mCherry (Figure S5A_1_). Similarly, germline excision of the STOP cassette shows a neuropil distribution (Figure S5B_2_) that closely resembles Synapsin (Figure S5B_3_) as revealed in the overlay (Figure S5B_4_). An overlay of the stack comprising Figure S5B is presented as a movie to enable visualization of individual slices (Fig S6). Some anti-OLLAS signal was observed in the absence of a GAL4 driver (Figure S5C_2_), thus indicating either the *FRT-STOP-FRT-6XOLLAS-vAChT* exhibits slight leak prior to STOP cassette excision or the anti-OLLAS antibody recognizes an endogenous Drosophila antigen.

Similar results were observed for *B2RT-STOP-B2RT-6XOLLAS-vAChT*. Pan-neuronal STOP cassette excision using *N-syb-GAL4* resulted in a neuropil distribution of 6XOLLAS-vAChT (Figure S5D_2_) that closely matches that of endogenous Synapsin (Figure S5D_3_), as indicated in the overlay (Figure S5D_4_), that is distinct from CD8-mCherry (Figure S5D_1_). Germline excision of the STOP cassette also reveals a neuropil distribution of 6XOLLAS-vAChT (Figure S5E_2_) nearly indistinguishable from Synapsin (Figure S5E_3_) as indicated in the overlay (Figure S5E_4_). Some anti-OLLAS signal was observed in the absence of a GAL4 driver (Figure S5F_2_), thus indicating either the *B2RT-STOP-B2RT-6XOLLAS-vAChT* exhibits slight leak prior to STOP cassette excision or the anti-OLLAS antibody recognizes one or more endogenous Drosophila antigens. To distinguish between these two possibilities, anti-OLLAS immunostaining was performed on a *yw* control brain. This revealed similar levels of background (Figure S5G_2_) as controls, thus indicating the anti-OLLAS antibody does recognize low levels of an endogenous Drosophila antigen, and that the observed signal in controls (Figure S5C_2_ and S5F_2_) is not due to leaky/constitutive expression of 6XOLLAS-vAChT prior to STOP cassette expression.

Both *FRT-STOP-FRT-7XMYC* and *B2RT-STOP-B2RT-7XMYC* are lethal in combination with the *vAChT^1^* allele before STOP cassette excision, but viable in combination with *vAChT^1^* after germline excision of the STOP cassette. Moreover, *FRT-7XMYC-vAChT* and *B2RT-6XOLLAS-vAChT* chromosomes with germline excisions of the STOP cassette are homozygous viable with no developmental delay or obvious behavioral abnormalities. These observations indicate both 7XMYC-vAChT and 6XOLLAS-vAChT are functional vesicular transporters of acetylcholine.

### Assessment of neurotransmitter and SV specificity of 7XMYC-vAChT in single neuron types

**Figure 3:**
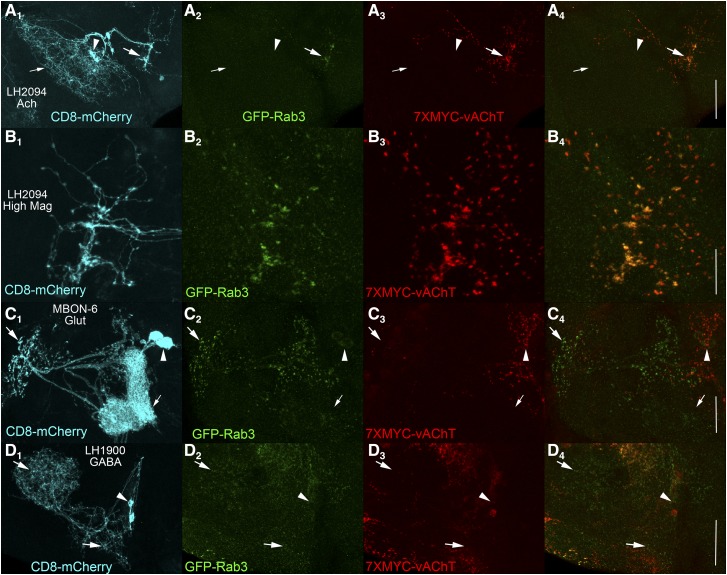
Assessment of synaptic vesicle and neurotransmitter specificity of *FRT-STOP-FRT-7XMYC-vAChT*. A) Cholinergic neuron LH2094. A_1_) CD8-mCherry; A_2_) GFP-Rab3; A_3_) 7XMYC-vAChT; A_4_) Overlay of GFP-Rab3 and 7XMYC-vAChT. Scale bar: 50μm. B) Higher resolution imaging of cholinergic neuron LH2094. B_1_) CD8-mCherry; B_2_) GFP-Rab3; B_3_) 7XMYC-vAChT; B_4_) overlay of GFP-Rab3 and 7XMYC-vAChT. 7XMYC-vAChT is expressed in present in LH2094 neurons and exhibits strong overlap with GFP-Rab3 in pre-synaptic terminals. Scale bar 20μm. C) Glutamatergic neuron MBON-6. C_1_) CD8-mCherry; C_2_) GFP-Rab3; C_3_) 7XMYC-vAChT; C_4_) overlay of GFP-Rab3 and 7XMYC-vAChT. GFP-Rab3 expression, but not 7XMYC-vAChT expression, is observed in MBON-6 neurons. Scale bar: 25μm. D) GABAergic neuron LH1900. D_1_) CD8-mCherry; D_2_) GFP-Rab3; D_3_) 7XMYC-vAChT; D_4_) overlay of GFP-Rab3 and 7XMYC-vAChT. GFP-Rab3 expression, but not 7XMYC-vAChT expression, is observed in LH1900 neurons. Scale bar 50μm. These results demonstrate the specificity of expression of *FRT-STOP-FRT-7XMYC-vAChT* for cholinergic and not glutamatergic or GABAergic neurons. Large arrows-pre-synaptic terminals; small arrows-dendrites; arrowheads-cell bodies.

To assess whether *FRT-STOP-FRT-7XMYC-vAChT* specifically expresses in cholinergic neurons, STOP cassette excision in neurons with known neurotransmitter usage previously determined using independent methods was performed using split-GAL4 drivers. As a positive control for cholinergic neurons, the split-GAL4 driver for lateral horn neuron LH2094 (Dolan *et al.* 2019) was chosen. Two additional markers were included in this and all subsequent analyses, the plasma membrane marker CD8-mCherry to visualize the entire neuron(s) of interest and the conditionally expressible neurotransmitter-independent SV marker *B2RT-STOP-B2RT-GFP-Rab3* (Williams *et al.* 2019) to visualize SV localization. The LH2094 neuron (Figure 3A_1_) is a highly polarized neuron with a large dendritic region (small arrow), cell bodies (arrowhead), and a small distinct region containing axon terminals (large arrow). Conditional expression of GFP-Rab3 in LH2094 neurons is highly restricted to the axon terminals (Figure 3A_2_) as is conditional expression of 7XMYC-vAChT (Figure 3A_3_). The coincident expression of 7XMYC-vAChT with GFP-Rab3 in LH 2094 neurons as shown in the overlay (Figure 3A_4_) demonstrates 7XMYC-vAChT is a reliable marker of cholinergic SVs. The observed expression of both GFP-Rab3 and 7XMYC-vAChT outside the LH2094 neuron is a common, previously observed phenomenon even with highly specific split-GAL4 drivers (Harris *et al.* 2015; Williams *et al.* 2019). This is a due to developmental expression of GAL4 in other neurons besides the ones the GAL4 driver may be restricted to during adulthood, as once excision of a STOP cassette occurs, it is permanent even if GAL4 is no longer expressed in those neurons in adults. Inclusion of the CD8-mCherry plasma membrane marker is thus critical for distinguishing conditional expression of GFP-Rab3 and 7XMYC-vAChT within the neurons of interest from expression in other neurons. Higher magnification images of LH2094 axon terminals reveal a near perfect overlap between GFP-Rab3 (Figure 3B_2_) and 7XMYC-vAChT (Figure 3B_3_) as evident in the overlay (Figure 3B_4_). An overlay of the stacks comprising Figure 3B are presented as a movie to enable visualization of individual slices (Fig S7).

Expression of 7XMYC-vAChT was assessed in the known glutamatergic neuron MBON-6 (Figure 3C_1_) (Aso *et al.* 2014). This neuron is also highly polarized with a prominent dendritic region encompassing a portion of the mushroom body (small arrow), cell bodies (arrowhead), and axon terminals (large arrow). Conditional expression of GFP-Rab3 in MBON-6 neurons reveals sites of SV release (Figure 3C_2_) but there is no corresponding expression of 7XMYC-vAChT (Figure 3C_3_). Background expression of 7XMYC-vAChT does not overlap with GFP-Rab3 (Figure 3C_4_), thus indicating 7XMYC-vAChT does not express in glutamatergic neurons.

7XMYC-vAChT expression was also evaluated in the known GABAergic neuron LH1900 (Figure 3D_1_) (Dolan *et al.* 2019). In this neuron cell bodies are easily identified (arrowhead), but axonic and dendritic regions intermingle and are not easily distinguished (large arrows). SVs distribute extensively throughout this neuron as indicated with GFP-Rab3 (Figure 3D_2_). 7XMYC-vAChT is not expressed in LH1900 neurons (Figure 3D_3_) as background expression of 7XMYC-vAChT does not overlap with GFP-Rab3 expression in LH1900 neurons (Figure 3D_4_). This result indicates 7XMYC-vAChT does not express in GABAergic neurons. Together these results demonstrate 7XMYC-vAChT expression is specific for cholinergic neurons and subcellular localization is specific for SVs based on near precise colocalization with GFP-Rab3.

### Assessment of neurotransmitter and SV specificity of 6XOLLAS-vAChT in single neuron types

**Figure 4:**
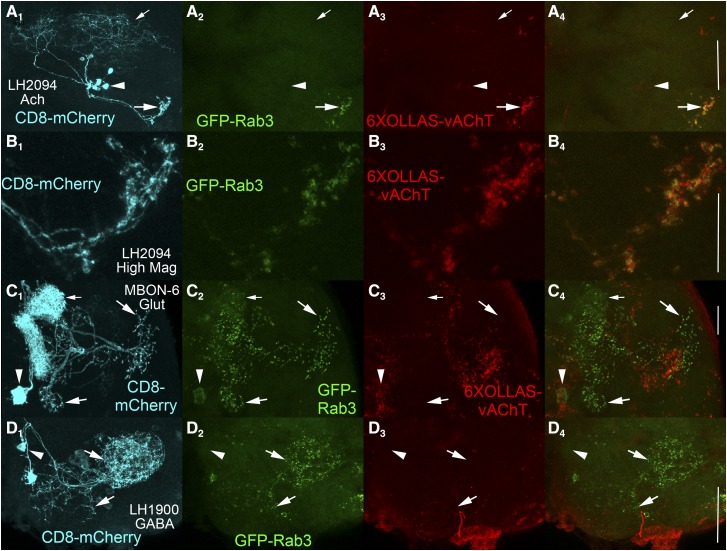
Assessment of synaptic vesicle and neurotransmitter specificity of *FRT-STOP-FRT-6XOLLAS-vAChT*. A) Cholinergic neuron LH2094. A_1_) CD8-mCherry; A_2_) GFP-Rab3; A_3_) 6XOLLAS-vAChT; A_4_) Overlay of GFP-Rab3 and 6XOLLAS-vAChT. Scale bar: 50μm. B) Higher resolution imaging of cholinergic neuron LH2094. B_1_) CD8-mCherry; B_2_) GFP-Rab3; B_3_) 6XOLLAS-vAChT; B_4_) overlay of GFP-Rab3 and 6XOLLAS-vAChT. 6XOLLAS-vAChT expression is present in LH2094 neurons and exhibits strong overlap with GFP-Rab3 in pre-synaptic terminals. Scale bar: 20μm. C) Glutamatergic neuron MBON-6. C_1_) CD8-mCherry; C_2_) GFP-Rab3; C_3_) 6XOLLAS-vAChT; C_4_) overlay of GFP-Rab3 and 6XOLLAS-vAChT. GFP-Rab3 expression, but not 6XOLLAS-vAChT expression, is observed in MBON-6 neurons. Scale bar: 25μm. D) GABAergic neuron LH1900. D_1_) CD8-mCherry; D_2_) GFP-Rab3; D_3_) 6XOLLAS-vAChT; D_4_) overlay of GFP-Rab3 and 6XOLLAS-vAChT. GFP-Rab3 expression, but not 6XOLLAS-vAChT expression, is observed in LH1900 neurons. Scale bar: 50μm. These results demonstrate the specificity of expression of *FRT-STOP-FRT-6XOLLAS-vAChT* for cholinergic neurons and not glutamatergic or GABAergic neurons. Large arrows-pre-synaptic terminals; small arrows-dendrites; arrowheads-cell bodies. Scale bar: 50μm.

A similar assessment for specificity of expression in cholinergic neurons was performed for *FRT-STOP-FRT-6XOLLAS-vAChT*. In the cholinergic neuron LH2094 (Figure 4A_1_), axon terminals are revealed by GFP-Rab3 localization (Figure 4A_2_). 6XOLLAS-vAChT is also expressed in LH2094 neurons (Figure 4A_3_) highly co-incident with GFP-Rab3 (Figure 4A_4_), thus demonstrating 6XOLLAS-vAChT is a reliable marker of SVs in cholinergic neurons. Higher magnification images of LH2094 axon terminals reveals a high degree of overlap between GFP-Rab3 (Figure 4B_2_) and 6XOLLAS-vAChT (Figure 4B_3_) as evidenced in the overlay (Figure 4B_4_). The stacks comprising Figure 4 are presented as a movie to enable visualization of individual slices (Fig S8).

Assessment of 6XOLLAS-vAChT expression in glutamatergic neuron MBON-6 (Figure 4C_1_) was also performed. Axon terminals were revealed by GFP-Rab3 localization (Figure 4C_2_). Although background expression of 6XOLLAS-vAChT was observed (Figure 4C_3_) it did not come from MBON-6 neurons as indicated by failure of overlap with GFP-Rab3 (Figure 4C_4_). This result indicates 6XOLLAS-vAChT does not express in glutamatergic neurons.

The GABAergic neuron LH1900 (Figure 4D_1_) was used to assess expression of 6XOLLAS-vAChT. Pre-synaptic terminals were indicated by GFP-Rab3 localization (Figure 4D_2_). Minimal background expression of 6XOLLAS-vAChT was observed (Figure 4D_3_), but no overlap was observed with GFP-Rab3 (Figure 4D_4_). This indicates 6XOLLAS-vAChT does not express in GABAergic neurons. Combined, these results establish 6XOLLAS-vAChT is specific for cholinergic neurons and subcellular localization is specific for SVs based on strong colocalization with GFP-Rab3.

### Cholinergic neurotransmitter phenotyping neurons of unknown neurotransmitter usage

**Figure 5:**
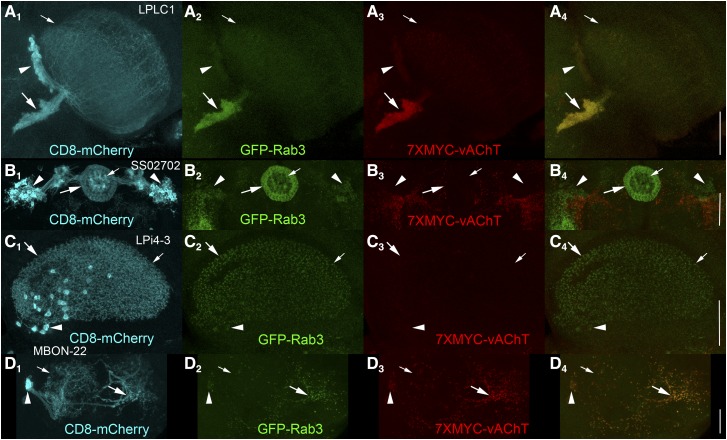
Cholinergic neurotransmitter phenotyping of neurons of unknown neurotransmitter usage using *FRT-STOP-FRT-7XMYC-vAChT*. A) Lobular plate/lobula column neuron LPLC1. A_1_) CD8-mCherry; A_2_) GFP-Rab3; A_3_) 7XMYC-vAChT; A_4_) overlay of GFP-Rab3 and 7XMYC-vAChT. 7XMYC-vAChT is expressed in the pre-synaptic terminals of LPLC1 neurons, thus indicating LPLC1 is a cholinergic neuron. Scale bar: 50μm. B) Ellipsoid body neuron SS02702. B_1_) CD8-mCherry; B_2_) GFP-Rab3; B_3_) 7XMYC-vAChT; B_4_) overlay of GFP-Rab3 and 7XMYC-vAChT. The absence of expression of 7XMYC-vAChT in SS02702 neurons suggests SS02702 neurons are not cholinergic. Scale bar: 50μm. C) Lobula plate intrinsic neuron Lpi4-3. C_1_) CD8-mCherry; C_2_) GFP-Rab3; C_3_) 7XMYC-vAChT; C_4_) overlay of GFP-Rab3 and 7XMYC-vAChT. The absence of expression of 7XMYC-vAChT in LPi4-3 neurons suggests Lpi4-3 neurons are not cholinergic. Scale bar: 50μm. D) Mushroom body output neuron MBON-22. D_1_) CD8-mCherry; D_2_) GFP-Rab3; D_3_) 7XMYC-vAChT; D_4_) overlay of GFP-Rab3 and 7XMYC-vAChT. 7XMYC-vAChT is expressed in the pre-synaptic terminals of MBON-22 neurons, thus indicating MBON-22 is a cholinergic neuron. Scale bar: 25μm.

To illustrate the utility of these conditional epitope tag-multimerized vAChT fly strains, four diverse neuron types of unknown neurotransmitter usage were neurotransmitter phenotyped for acetylcholine using *FRT-STOP-FRT-7XMYC-vAChT*. The first of these, lobular plate/lobular columnar 1 (LPLC1) neurons (Figure 5A_1_) (Wu *et al.* 2016), have a large dendritic field in the lobula (small arrow), cell bodies on the border between the optic lobe and central brain (arrowhead), and axonal projections into the central brain (large arrow). The vast majority of GFP-Rab3 localizes to the axonal projections in the central brain (Figure 5A_2_). 7XMYC-vAChT expression is also observed in LPLC1 neurons (Figure 5A_3_) and distributes coincident with GFP-Rab3 as demonstrated in the overlay (Figure 5A_4_), thus establishing LPLC1 as a cholinergic neuron. The small fraction of both GFP-Rab3 and 7XMYC-vAChT that distributes to the presumptive dendritic region of LPLC1 neurons suggests this region is not exclusively dendritic but also transmits information via acetylcholine release.

The ellipsoid body neuron type represented by split-GAL4 driver SS02702 (Figure 5B_1_) (Robie *et al.* 2017) has distal cell bodies (arrowheads) that project medially to form two concentric rings in the center of the brain. The localization of GFP-Rab3 nearly exclusively to these rings (Figure 5B_2_) indicates these regions of the neuron as the sites of SV release. The stronger signal in the outer ring (large arrow) relative to the inner ring (small arrow) suggests the outer ring is predominantly axonal although both rings may be a mixture of both axons and dendrites. No expression of 7XMYC-vAChT was observed in either ring (Figure 5B_3_) and only GFP-Rab3 signal is present in the rings in the overlay (Figure 5B_4_) even though background expression of 7XMYC-vAChT is apparent in non-SS02702 neurons. This result strongly suggests SS02702 ellipsoid body neurons are not cholinergic.

The lobula plate intrinsic neuron 4-3 (LPi4-3) (Klapoetke *et al.* 2017) has cell bodies (arrow) and an intermingled axonal/dendritic region (small and large arrows) intrinsic to the lobula plate. GFP-Rab3 exhibits a nearly uniform, punctate distribution throughout this region (Figure 5C_2_). No corresponding expression of 7XMYC-vAChT was observed in LPi4-3 neurons (Figure 5C_3_, 5C_4_), strongly suggesting they are not cholinergic. This result is consistent with functional studies of LPi4-3 in which its optogenetic activation inhibited the activity of downstream LPLC2 neurons (Klapoetke *et al.* 2017). If LPi4-3 were cholinergic, optogenetic activation would have been expected to excite LPLC2 neurons as acetylcholine is well established as an excitatory neurotransmitter.

Mushroom body output neuron 22 (MBON-22) (Figure 5D_1_) (Aso *et al.* 2014) has distal cell bodies (arrowhead), with a large spherical region (small arrow) that is distinct from a more proximal region near the center of the brain (large arrow). The absence of GFP-Rab3 in the large spherical region establishes this region as dendritic and its presence in the more proximal region establishes it as axonal (Figure 5D_2_). 7XMYC-vAChT expression is also observed in MBON-22 neurons (Figure 5D_3_) and exhibits tight colocalization with GFP-Rab3 in the overlay (Figure 5D_4_). This result demonstrates MBON-22 is a cholinergic neuron.

### Sensitivity comparison of 7XMYC-vAChT, 6XOLLAS-vAChT, and HA-vAChT

**Figure 6:**
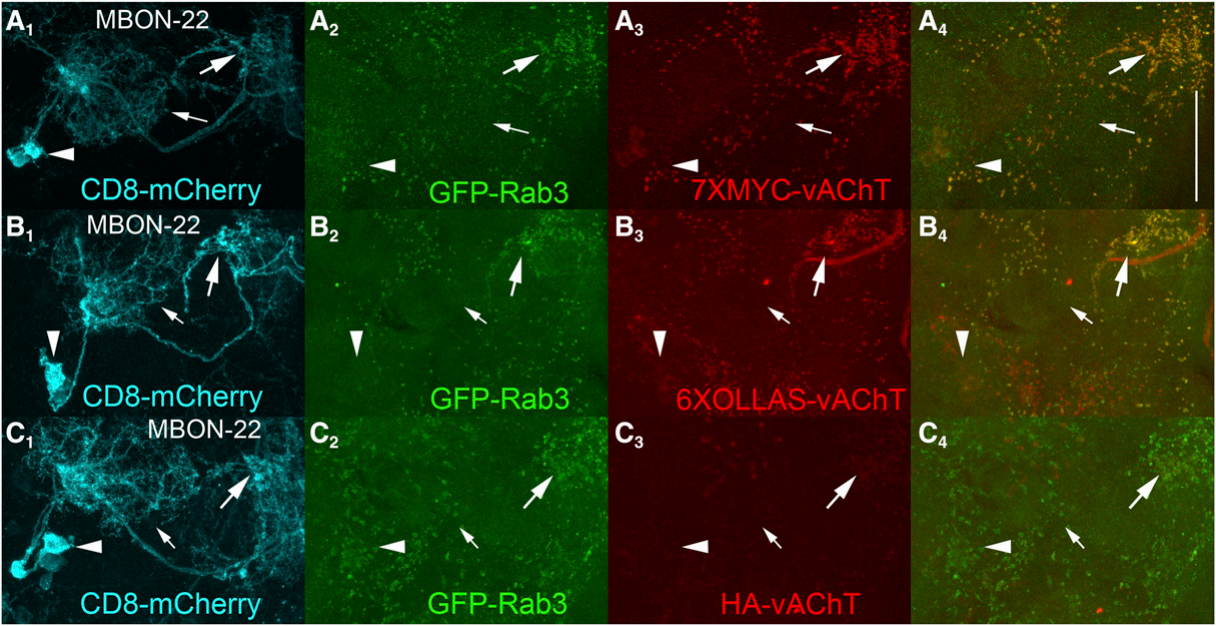
Relative sensitivity of conditional 7XMYC-vAChT, 6XOLLAS-vAChT, and HA-vAChT in cholinergic MBON-22 neurons. A_1_) CD8-mCherry; A_2_) GFP-Rab3; A_3_) 7XMYC-vAChT. B_1_) CD8-mCherry; B_2_) GFP-Rab3; B_3_) 6XOLLAS-vAChT. C_1_) CD8-mCherry; C_2_) GFP-Rab3; C_3_) HA-vAChT. Signal intensity is significantly higher for 7XMYC-vAChT and 6XOLLAS-vAChT as compared to HA-vAChT. Immunostaining, image acquisition, and image processing was identical between all three genotypes. Large arrows-pre-synaptic terminals; small arrows-dendrites; arrowheads-cell bodies. Scale bar: 50μm.

To qualitatively assess the relative sensitivity of detection of 7XMYC-vAChT, 6XOLLAS-vAChT, and HA-vAChT, each was conditionally expressed in the cholinergic neuron MBON-22. Images for each genotype were acquired and processed identically for this assessment. Strong signal from 7XMYC-vAChT (Figure 6A_3_, large arrow) coincident with GFP-Rab3 (Figure 6A_2_, 6A_4_) was observed in the axonal region of MBON-22 neurons. Similarly, strong signal from 6XOLLAS-vAChT (Figure 6B_3_, large arrow) coincident with GFP-Rab3 (Figure 6B_2_, 6B_4_) was observed in the axonal region of MBON-22 neurons. Noticeably weaker signal was observed from HA-vAChT (Figure 6C_3_, large arrow) coincident with GFP-Rab3 (Figure 6C_2_, 6C_4_) in the axonal region of MBON-22 neurons. 7XMYC-vAChT and 6XOLLAS-vAChT signal intensity is thus qualitatively similar, while HA-vAChT signal is considerably weaker in MBON-22 neurons. This result is as expected since the 7XMYC-vAChT contains seven copies of the MYC epitope, 6XOLLAS-vAChT contains six copies of the OLLAS epitope, and HA-vAChT contains only one copy of the HA epitope.

## Discussion

A simple strategy discovered by serendipity for multimerizing DNA repeats is described that utilizes the Gibson cloning reaction. The effectiveness of this strategy was demonstrated using coding sequences for the commonly used MYC and OLLAS epitope tags. Attempts were made to optimize the method for maximum repeat number by varying both substrate quantity and reaction time. Although these attempts at optimization only showed minimal differences that were not statistically significant, they did demonstrate that seven copies of the MYC repeat could be recovered with relative ease and recovery of at least ten copies is possible even though the original design was only for two copies. The same repeat multimerization phenomenon was observed with the OLLAS epitope tag coding sequence where six copies of the repeat were recovered. These results indicate the strategy should be generally applicable for multimerizing any non-internally repetitive DNA sequence in the same size range since the MYC and OLLAS coding sequences share no sequence similarity. The utility of this method of generating DNA repeats is noteworthy because commercial DNA synthesis companies often refuse to even attempt synthesis of the coding sequences of more than two or three tandem copies of epitope tag repeats due to high levels of complexity. Thus, there is no easy alternative for generating DNA repeats of the number and size of which this method has been demonstrated capable.

### Mechanism of DNA repeat multimerization

A possible mechanism for Gibson reaction-mediated DNA multimerization is presented (Figures 2C-I) in which repeat multimerization occurs as a result of successive rounds of DNA synthesis and alternative annealing configurations prior to DNA ligation. Regardless of the extent to which this proposed mechanism is correct, it seems highly likely that once ligation occurs the possibility of further multimerization of repeat number is lost. Thus, one potential strategy for enhancing the multimerization phenomena would be to reduce the concentration of DNA ligase relative to the 5′-3′ exonuclease and DNA polymerase enzymes. This is currently not feasible given the ratios of the three enzymes in commercially available Gibson formulations is fixed. However, perhaps a variant version of Gibson reactions inspired by these findings could become commercially available in the future that is specifically intended for maximizing the copy number of DNA repeats in which the amount of the DNA ligase is reduced relative to the other two Gibson reaction enzymes. The proposed mechanism of multimerization resulting from alternative annealing configurations of the repeats would predict that the larger the size of the repeat, the less effective the repeat multimerization because larger repeats should be more stable due to the larger number of hydrogen bonds and thus denature and rearrange at lower frequency. Conversely, the proposed multimerization mechanism would predict that smaller repeats would multimerize with greater efficiency.

The results reported above demonstrate that the strategy is effective for multimerizing DNA sequence repeats of at least 56 base pairs as the 14 amino acid OLLAS epitope tag sequence is encoded by 42bp and the 12 bp coding sequence for the four amino acid GGSG flexible linker were included between each OLLAS repeat (the MYC repeat totaled 42bp, 30bp of MYC epitope coding sequence and 12bp of linker coding sequence). As OLLAS at 14 amino acids is among the largest of the commonly used epitope tag sequences, the method should be applicable for multimerizing the coding sequence of any currently available epitope tag. However, the method should be useful for multimerizing other types of DNA sequences as well, such as regulatory regions/transcription factor binding sites.

### Application of the epitope tag multimerization method to Drosophila vAChT

Proof of principle of the practical utility of the DNA repeat multimerization strategy for epitope tags was demonstrated by multimerizing seven copies of the MYC or six copies of the OLLAS epitope tag coding sequences fused to the amino terminus of Drosophila vAChT. Conditionally expressible versions of these vAChT variants were subsequently integrated into the endogenous vAChT locus via CRISPR/Cas9 genome editing. Four variants were generated utilizing both *FRT* and *B2RT*-flanked STOP cassettes and 7XMYC or 6XOLLAS tags. All four variants were shown to be conditionally expressible (*i.e.*, with no detectable leak/constitutive expression). Both the 7XMYC-vAChT and 6XOLLAS-vAChT were demonstrated to express specifically in cholinergic neurons, but not glutamatergic or GABAergic neurons, and to exhibit robust signal in co-localizing with the general SV marker GFP-Rab3 in cholinergic neurons. The advantage of the enhanced sensitivity of 7XMYC-vAChT and 6XOLLAS-vAChT as compared to (1X) HA-vAChT was also demonstrated, as was the utility of *FRT-STOP-FRT-7XMYC-vAChT* for cholinergic neurotransmitter phenotyping of single neuron types. These four, conditional epitope-tagged vAChT variants will thus be valuable tools as cholinergic SV markers and for neurotransmitter phenotyping.

### Comparison to alternatives for enhancing protein detection sensitivity

For enhancing the sensitivity of protein detection this epitope multimerization strategy is an alternative to the spaghetti monster (sm) proteins that typically contain 10 copies of commonly used epitope tags integrated into GFP (Viswanathan *et al.* 2015). Although the method described here routinely produces slightly fewer copies of epitope tags at six or seven, it does occasionally generate 10 copies of epitope tag sequences, and thus can equal the sensitivity enhancement of sm proteins. Potential advantages of this strategy include: 1) smaller coding sequences enhance cloning efficiency; 2) the multimerized epitope tags are less likely to disrupt the function and/or localization of the protein of interest (especially if flexible linkers are added between the repeats as was done here) as compared to the sm proteins since GFP has a rigid structure; 3) it is possible to include immunostaining for GFP as part of experiments with epitope multimerized proteins but not with sm proteins since sm proteins are recognized by GFP antibodies; and 4) it can be used with any epitope tag and not just those available with existing sm proteins.
